# Mucopolysaccharidosis and Autophagy: Controversies on the Contribution of the Process to the Pathogenesis and Possible Therapeutic Applications

**DOI:** 10.1007/s12017-019-08559-1

**Published:** 2019-08-01

**Authors:** Karolina Pierzynowska, Lidia Gaffke, Magdalena Podlacha, Joanna Brokowska, Grzegorz Węgrzyn

**Affiliations:** Department of Molecular Biology, Faculty of Biology, University of Gdańsk, Wita Stwosza 59, 80-308 Gdańsk, Poland

**Keywords:** Mucopolysaccharidosis, Autophagy, Glycosaminoglycans, Genistein, Trehalose

## Abstract

Mucopolysaccharidosis (MPS) consists of a group of 11 enzymatic defects which result in accumulation of undegraded glycosaminoglycans (GAG) in lysosomes. MPS is a severe metabolic disease for which only bone marrow/hematopoietic stem cell transplantation and enzyme replacement therapy are current therapeutic options. However, they are available for only a few of MPS types, and are ineffective in treatment of central nervous system. Recent studies indicated that the autophagy process can be impaired in MPS, but various contradictory conclusions have been published in this matter. Nevertheless, stimulation of autophagy has been proposed as a potential therapeutic option for MPS, and very recent results suggest that such approach might be effective in improving MPS symptoms. Still the mechanisms of autophagy changes in MPS are not clear, and efficiency of autophagy activation in clearing the storage material requires further investigation. These problems are summarized and discussed in this review.

## Introduction

**Fig. 1:**
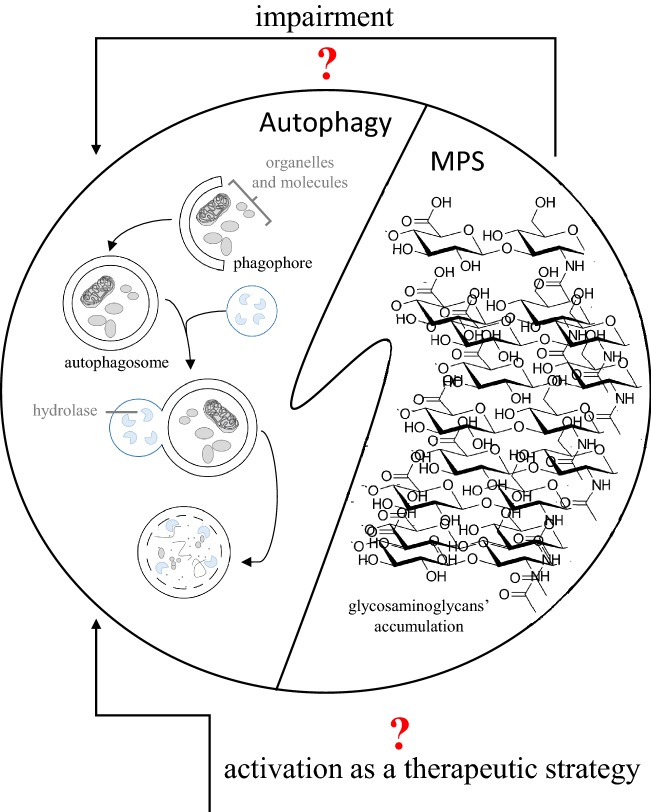
The scheme of the possible influence of mucopolysaccharidosis (MPS) on autophagy, and a proposal for autophagy activation as a therapeutic strategy for MPS treatment. Mucopoilysaccharidosis (MPS) is a group of inherited metabolic diseases caused by impaired degradation and resultant accumulation of glycosaminoglycans in lysosomes (right panel). This accumulation leads to dysfunction of lysosomes and possible (question mark) impairment of the autophagy process (left panel). Activation of autophagy can be considered (question mark) as a therapeutic strategy for MPS, as enhanced degradation of primary and/or secondary storage material might restore cellular functions. This approach could be particularly useful in treatment of neuronopathic forms of MPS, where no effective therapy for central nervous system symptoms is currently available

Mucopolysaccharidosis (MPS) is a common name for the group of lysosomal storage diseases (LSD), characterized by accumulation of undegraded glycosaminoglycans (GAGs) (Fig. 1). These complex sugars are stored in lysosomes of patient’s cells due to genetic defects resulting in dysfunction of enzymes involved in GAG degradation. Depending on the nature of the lacking or defective enzyme, 11 types and subtypes of MPS are distinguished. GAG storage leads to various secondary and tertiary changes in cells, tissues, and organs, which cause severe symptoms in patients. Although enzyme replacement therapy is currently available for some MPS types (MPS I, MPS II, MPS IVA, MPS VI, and MPS VII), this treatment can be effective in alleviating somatic symptoms, but not those occurring in central nervous system, as the therapeutic enzyme cannot cross the blood–brain barrier. Therefore, there are various attempts to develop other therapies for MPS, particularly for the types in which neurodegeneration is a major problem (MPS I, MPS II, MPS IIIA, MPS IIIB, MPS IIIC, MPS IIID, MPS VII, and MPS IX). A comprehensive review on MPS, including molecular mechanisms of the disease, clinical features, diagnostic procedures, and currently available and potential therapeutic options, can be found in the recently published book (Tomatsu et al. 2018).

The lack of effective therapy for MPS which would correct all the disease symptoms (for a review see Tomatsu et al. 2018) stimulates studies on development of novel therapeutic options. Nevertheless, development of any effective strategy to cure MPS must be based on detailed understanding of molecular mechanisms of this disease. Since degradation of GAGs occurs in lysosomes, elucidation of all aspects of impairment of functions of these organelles appears to be a key point to find a truly effective therapy for MPS.

Autophagy is the process of degradation of various macromolecules which depends on the function of lysosomal apparatus (Meijer and Codogno 2007). The characteristic feature of this process is engulfment of the organelles, structures or molecules to be degraded by an intracellular membrane, thus, formation of autophagosome, and its fusion with lysosome(s). This allows the lysosomal system to degrade specifically designed biological material which is no longer required in the cell (Fig. 1). Three major types of autophagy: microautophagy, macroautophagy, and chaperone-dependent autophagy, are generally known (Cuervo 2004). Macroautophagy is the most common type of this process, and requires fusion of autophagosome with lysosome. In the case of microautophagy, invagination of lysosomal membrane allows sequestration of a cytoplasm fragment directly by the lysosome (Mijaljica et al. 2011). In chaperone-dependent autophagy, the Hsp70 class chaperones form complexes with substrate molecules that are transported into lysosomes and degraded there (Kaushik et al. 2011).

Since autophagy causes degradation of unnecessary or unwanted molecules, modulation of this process was proposed to be employed in various diseases (Zhan et al. 2018; Bonam et al. 2018; Bishop and Bradshaw 2018; Jakovljevic et al. 2018), and its stimulation has been considered as a possible therapeutic approach for treatment of diseases that are caused by accumulation of pathogenic macromolecules (Boland et al. 2018; Xin et al. 2018). These include misfolded protein aggregates which are primary pathogenic agents in different diseases, including the most common severe neurodegenerative disorders, like Alzheimer’s disease, Parkinson’s disease, and Huntington’s disease. In fact, activation of autophagy has recently been proposed as a promising strategy for development of drugs for such neurodegenerative diseases (Pierzynowska et al. 2018a, 2018b). There are some molecules that are not only autophagy stimulators, but also can cross the blood–brain barrier and appear to be safe in a long-term treatment of patients, which make them promising candidates for drugs, as discussed recently (Pierzynowska et al. 2018a). However, the remaining question is whether stimulation of autophagy can be considered as a therapeutic approach in diseases caused by lysosomal dysfunctions? First, is autophagy changed in cells of patients suffering from MPS? Second, could autophagy activation help to degrade storage material (either primary or secondary) which is already accumulated in lysosomes due to dysfunction of one of lysosomal enzymes? Third, could stimulation of autophagy lead to efficient degradation of accumulated compounds other than proteins? In this mini-review, we will focus on MPS as examples of LSD (other LSDs will only be mentioned shortly, though there is a substantial literature on autophagy in these diseases, but our aim was to concentrate on MPS), and discuss both changes in the autophagy process occurring in MPS cells and therapeutic potential of autophagy stimulators.

## Changes in the Autophagy Process in MPS Cells

Since lysosomal storage impairs functions of these organelles, and autophagy is a process which requires activities of lysosomes, the question whether MPS or other LSDs result in changes in autophagy appears reasonable. However, the first reports on studies addressing this problem were published relatively recently, some 10 years ago. It was demonstrated that in two LSDs, including MPS IIIA, the autophagosome–lysosome fusion is impaired (Settembre et al. 2008a). On the other hand, studies with autophagy stimulator (rapamycin) and inhibitor (bafilomycin A1) suggested that the block of this process was not complete in tested cells (Settembre et al. 2008a). Therefore, it was postulated that LSDs, including MPS, are ‘autophagy disorders.’ Since accumulation of protein aggregates and dysfunctional mitochondria was also observed, it was suggested that some pathomechanisms of LSD (MPS) and late-onset neurodegenerative diseases (like Alzheimer’s disease) might by common (Settembre et al. 2008b; Ballabio 2009).

If we focus on MPS, it is necessary to mention that similar observations were made when fibroblasts from MPS VI patients were studied. The autophagy process was partially blocked in these cells, while transfer of the wild-type gene to MPS VI cells resulted in correction of autophagy (Tessitore et al. 2009). It was concluded that GAG accumulation impairs lysosomal functions required to support autophagic degradation of various compounds (Tessitore et al. 2009). Such suggestion was corroborated by other studies (Karnati et al. 2016). In the MPS IIIC mouse model, increased levels of the LC3-II protein, the markers of autophagy, were detected (Pshezhetsky 2016). Such results may suggest either enhanced autophagosomal genesis or decreased autophagic flux, but no experiments were performed to distinguish between these two alternatives. Changes in expression of autophagy-related genes, coding for Atg1 and Atg18 proteins, were reported recently in MPS IIIA (Webber et al. 2018). Impaired autophagy has been observed also in MPS II (Fiorenza et al. 2018), as well as in MPS VII, where inhibition of the autophagic flux was demonstrated (Bartolomeo et al. 2017).

Intriguingly, opposite conclusions were presented by other groups. Based on MPS I mice studies, enhanced autophagy has been suggested (Woloszynek et al. 2009). In the same MPS type, highly elevated levels of expression of autophagy-related genes have been determined on the basis on transcriptomic and Gene Ontology analyses, which led to conclusion about enhanced autophagy in this disease (Swaroop et al. 2018).

Contrary to both groups of conclusions mentioned above, normal functions of the autophagy pathway were reported in MPS IIIB (Vitry et al. 2010). Accordingly, no alterations in autophagy could be detected in MPS I fibroblasts (Viana et al. 2017).

An interesting link between MPS and autophagy has been discovered recently (Kondo et al. 2017). A specific mutation in the *VPS33A* gene (c.1492C > T; p.Arg498Trp) was found to cause extremely high levels of heparan sulfate (one of GAGs) in plasma and urine of patients who developed severe MPS-like symptoms. Thus, it was proposed to classify this disorder as a new MPS type. Intriguingly, the VPS33A protein is involved in autophagy, while the c.1492C > T mutation did not alter this process (Kondo et al. 2017).

Analysis of the results of studies on the autophagy process in MPS, published during last 10 years and summarized above, indicated that there are contradictory conclusions on the efficiency of autophagy in this disease. Some groups demonstrated impaired autophagy in MPS cells, other authors concluded about enhancement of this process in MPS, while the third group reported no changes in autophagy, relative to control cells. What could be the reason for such discrepancy in results and conclusions? First, one should note that experiments were performed with cells and/or animals representing different MPS types. In each MPS type, different enzyme is deficient; thus, various kinds of GAGs are accumulated. Therefore, one cannot exclude that autophagy is differentially affected in various MPS types. Only studies performed simultaneously with all types of MPS might determine whether autophagy is similarly or differentially affected in the presence of accumulation of different GAGs. On the other hand, one should remember that interpretation of results of experiments designed to test autophagy efficiency is not always straight forward. For example, elevated levels of the autophagy marker, the LC3 protein form II (LC3-II) might indicate either enhanced autophagosome formation or decreased autophagic flux. In the latter case, the autophagy process is impaired rather than stimulated. Therefore, we suggest that discrepancies in published conclusions on the efficiency of autophagy in MPS could arise from various interpretations of obtained results. Despite these differences, it appears that in most cases, significant changes in autophagy were found in MPS cells which indicate that this process in affected by accumulation of GAGs. According to the arguments presented above, we favor the hypothesis that autophagy is generally impaired in MPS, while opposite conclusions might arise from ambiguous interpretation of the results and/or from considering only gene-expression patterns rather than actual changes in cellular processes. Nevertheless, we cannot exclude that autophagy might be differentially affected in various types or subtypes of MPS.

## Can Stimulation of Autophagy be Considered as a Potential Therapy for MPS?

Since autophagy leads to degradation of accumulated compounds in cells, one might assume that stimulation of this process could be assessed as a potential strategy to develop novel treatments for diseases caused by storage of various macromolecules. Such a strategy has been proposed, and has been discussed recently (Pierzynowska et al. 2018a). It is worth noting that since storage-based disorders are usually either inherited or neurodegenerative diseases (or both), a potential drug should not only induce autophagy efficiently (though not too efficiently, to avoid auto-destruction of the cell), but also be safe in a long-term use. However, it appears that the list of known autophagy inductors revealing both these properties is relatively short (Pierzynowska et al. 2018a).

In the case of MPS, additional doubts might be raised. MPS are lysosomal disorders, caused by dysfunction of one of lysosomal enzymes; thus, one can ask if stimulation of defective lysosomal system might be profitable. On the other hand, if the conclusion that autophagy may be stimulated in MPS is correct, then further activation of this process would be dangerous for patients rather than providing benefits. Contrary to such a prediction, it appears obvious that stimulation of lysosomes in MPS should result in the elevation of residual activity of the deficient enzyme, more effective degradation of GAGs, and improvement of cellular functions. Moreover, it is important to note that secondary storage of various compounds (including glycosphingolipids, proteins, and others) occurs in MPS, having an important role in the development of various symptoms, including neurodegeneration. Therefore, one might predict that even reduction of only secondary storage material should also be beneficial for MPS patients.

The first indication that enhanced lysosomal biogenesis might be profitable for MPS patients came from studies on genistein, a natural isoflavone, which was previously demonstrated to impair synthesis of GAG, thus, being considered in substrate reduction therapy for MPS (Piotrowska et al. 2006). However, further studies indicated that this isoflavone not only interferes with GAG production due to inhibition of epidermal growth factor receptor (EGFR) (Jakóbkiewicz-Banecka et al. 2009), but also stimulates expression of vast majority of genes coding for lysosomal proteins (Moskot et al. 2014). This stimulation is due to enhanced synthesis of transcription factor EB (TFEB), the master regulator of lysosomal biogenesis, as well as its activation due to inhibition of activity of the mTOR kinase (Moskot et al. 2014). Therefore, genistein can be considered an autophagy stimulator which has been demonstrated experimentally (Pierzynowska et al. 2018b).

Despite indication that genistein can activate autophagy, it was not known if such activation can be of therapeutic potential in MPS. However, very recent studies demonstrated that trehalose, another autophagy stimulator, could improve symptoms of MPS IIIB mice (Lotfi et al. 2018). Trehalose-treated animals revealed longer life span, less hyperactivity and anxiety, improved vision, and reduced neuroinflammation, relative to untreated controls. Importantly, more efficient clearance of autophagic vacuoles was observed during trehalose treatment, which occurred together with activation of the TFEB-mediated stimulation of transcription of genes involved in lysosomal biogenesis (Lotfi et al. 2018). These results demonstrated that induction of autophagy can be profitable for organisms suffering from MPS in in vivo studies. This observation strongly suggests that autophagy is impaired rather than activated in MPS, at least in the MPS IIIB subtype, and stimulation of this process can result in enhancement of the autophagic system, leading to clearance of the storage material.

## Concluding Remarks

Recent studies indicated that the autophagy process may be impaired in MPS, and that its stimulation could be a promising therapeutic approach in this group of LSD (summarized in Fig. 1). However, many questions remain to be answered. First, is autophagy defective in all or only some types of MPS? Second, can stimulation of autophagy lead to clearance of the primary storage material (GAGs, stored due to deficiency of specific enzyme) or only secondary storage compounds? Third, could MPS patients with all types of mutations, or only those with residual activity of the deficient enzyme, benefit from enhanced autophagy (considering that if the enzyme is totally missing, induction of the lysosomal system might be ineffective anyway)? Fourth, which autophagy stimulator is the most effective in reducing storage and symptoms of MPS? Fifth, which autophagy stimulator is safe enough to be used for treatment of patients in a life-long therapy, which is necessary in MPS? Definitely, further studies should address these questions, and investigate stimulation of autophagy as a promising therapeutic strategy for MPS.
